# Nasal Carriage of Methicillin-Resistant *Staphylococcus aureus* among Health Care Workers in Tertiary and Regional Hospitals in Dar es Salam, Tanzania

**DOI:** 10.1155/2018/5058390

**Published:** 2018-09-10

**Authors:** Agricola Joachim, Sabrina J. Moyo, Lillian Nkinda, Mtebe Majigo, Sima Rugarabamu, Elizabeth G. Mkashabani, Elia J. Mmbaga, Naboth Mbembati, Said Aboud, Eligius F. Lyamuya

**Affiliations:** ^1^Department of Microbiology and Immunology, Muhimbili University of Health and Allied Sciences, Dar Es Salaam, Tanzania; ^2^Department of Clinical Science, University of Bergen, Bergen, Norway; ^3^Department of Epidemiology and Biostatistics, Muhimbili University of Health and Allied Sciences, Dar Es Salaam, Tanzania; ^4^Department of Surgery, Muhimbili University of Health and Allied Sciences, Dar Es Salaam, Tanzania

## Abstract

Methicillin-resistant *Staphylococcus aureus* (MRSA) among health care workers (HCWs) increases the risk of spreading the organism in hospital settings. A cross-sectional study was conducted between June and October 2016 among HCWs in tertiary and regional hospitals in Dar es Salaam, Tanzania, to determine the MRSA nasal carriage rate. Nasal swabs were collected from HCWs and cultured on mannitol salt agar. *S. aureus* was identified based on colonial morphology, Gram staining, catalase, coagulase, and DNase test results. MRSA was detected using the cefoxitin disk. Among 379 HCWs enrolled, 157/379 (41.4%) were colonized with *S. aureus*, of whom 59 (37.6%) were MRSA carriers giving an overall prevalence of 59/379 (15.6%). MRSA carriage was high among HCWs in Temeke (56.9%) and Amana (37.5%) regional hospitals. A high proportion of MRSA carriage was detected among nurses (35, 45.5%). MRSA isolates showed high resistance toward kanamycin (83.7%), gentamicin (83.1%), ciprofloxacin (71.2%), and trimethoprim-sulphamethoxazole (46.8%) compared to methicillin-sensitive *S. aureus* isolates (*p* ≤ 0.001). In conclusion, we found a high nasal carriage of MRSA and resistance to commonly prescribed antimicrobial agents among HCWs. Implementation of infection control measures including contact precautions, urgent reporting of MRSA laboratory results, and routine MRSA screening of HCWs is highly needed to reduce MRSA spreading.

## 1. Introduction

Methicillin-resistant *Staphylococcus aureus* (MRSA) is recognized as a major nosocomial pathogen that causes severe morbidity and mortality worldwide [1, 2]. MRSA prevalence is high among health care workers (HCWs), and there is a dynamic spread of strains across the globe. The prevalence varies from hospital to hospital in various countries, with high rates (32–52%) reported mainly in the developing countries [3, 4].

MRSA carriage among HCWs can render other measures of infection control ineffective [5, 6]. In most cases, colonized HCWs are generally asymptomatic, but they can be a potential reservoir of infection for susceptible patients. Moreover, it has been reported that HCWs have been the source of MRSA outbreaks in several hospital settings [6]. The spread of MRSA strains in resource-limited settings may cause devastating consequences due to lack of adequate facilities for laboratory detection and patient management [7]. MRSA carriage is an important predisposing factor for developing MRSA infection [8], and this increases the cost of patient care. Given the impact of MRSA infection, early identification of colonized patients and HCWs and implementing control measures such as decolonization of high-risk patients or those undergoing surgical procedures can minimize the chances of developing MRSA infection [9, 10]. A study by Sai et al reported an efficacy of decolonization of 73% when considering patients with carriage limited to nasal cavities [11]. Several studies conducted in Tanzania have shown persistently high rates of antimicrobial resistance, with *S. aureus* being reported as a predominant pathogen in patients with surgical site infections [12–14]. These studies on MRSA infection among hospitalized patients have also revealed an increasing trend of MRSA prevalence from 0.4% in 1999 to 28% in 2010 [15, 16]. We have recently reported an MRSA prevalence of 8.5% on admission among patients attending two regional hospitals in Dar es Salaam [17]. There is paucity of data on MRSA carriage among HCWs in Tanzania, with only two studies reporting a prevalence of 2.1% in Dar es Salaam and 0.3% in Mwanza regions [18, 19]. Thus, the aim of this study was to determine the rate of MRSA nasal carriage among HCWs at tertiary and regional hospitals in Dar es Salaam, Tanzania, in order to obtain objective findings that can inform the development of evidence-based control and preventive measures.

## 2. Methods

### 2.1. Study Design and Sampling Procedures

This is a hospital-based cross-sectional study conducted among HCWs at Muhimbili National Hospital (MNH), Ocean Road Cancer Institute (ORCI), and two regional hospitals, namely, Temeke and Amana, in Dar es Salaam, Tanzania. A total of 379 consenting HCWs were recruited in the study between June and October 2016. HCWs were recruited from medical wards (internal medicine, paediatric, and oncology), surgical wards (surgery, dental, obstetric, and gynecology), and intensive care unit (ICU). HCWs who were using antibiotics at the time of recruitment were excluded from participating. A structured questionnaire was used to collect sociodemographic information and factors associated with MRSA. Nasal specimens were collected from the anterior nares using sterile cotton swabs (Improswab, Guangzhou, China) moistened with normal saline. Both nostrils were sampled one at a time using the same swab by rotating gently against the inner surface. The swabs were placed in Stuart's transport media (in-house made) (Oxoid, Basingstoke, UK) and transported to the Microbiology and Immunology Laboratory at Muhimbili University of Health and Allied Sciences (MUHAS) for processing within eight hours of collection.

### 2.2. Laboratory Procedures

Nasal swabs were inoculated onto mannitol salt agar plates (Oxoid, Basingstoke, UK) for *S. aureus* isolation. The plates were incubated at 37°C and examined for growth after 24–48 hours. *S. aureus* was initially screened based on the presence of golden yellowish or creamy white colonies on mannitol salt agar and subcultured on nutrient agar (NA). The isolates from NA were further identified by Gram staining reaction and catalase test reaction and confirmed phenotypically by coagulase and/or DNase tests.

The antimicrobial susceptibility testing was carried out by using Kirby–Bauer's disk diffusion method as per Clinical and Laboratory Standards Institute (CLSI) 2015 guidelines [20]. The following standard antibiotic disks (Oxoid, Basingstoke, UK) were used: penicillin G (10 U), trimethoprim-sulphamethoxazole (1.25/23.75 *μ*g), gentamicin (10 *μ*g), kanamycin (30 *μ*g), erythromycin (15 *μ*g), clindamycin (2 *μ*g), ciprofloxacin (5 *μ*g), linezolid (30 *μ*g), and mupirocin (5 *μ*g). A standard inoculum was prepared by direct colony suspension in saline and compared with 0.5 McFarland standard turbidity and inoculated on the Muller-Hinton agar plate (Oxoid, Basingstoke, UK). The plates were incubated at 33–35°C for 16–18 hours. The results were interpreted according to the CLSI guidelines [20]. MRSA detection was done using cefoxitin disks (30 *μ*g) (Oxoid, Basingstoke, UK) according to CLSI 2015 guidelines. All *S. aureus* isolates resistant to cefoxitin were considered as MRSA. An inhibition zone of 21 mm or less around the cefoxitin disk indicated MRSA. *S. aureus* ATCC 25923 was used for quality control. In addition, clindamycin-inducible resistance was also tested by the D test as per CLSI guidelines [20]. Briefly, the erythromycin (15 *μ*g) disk was placed at a distance of 20 mm (edge to edge) from the clindamycin (2 *μ*g) disk on a Mueller-Hinton agar plate. After overnight incubation, the plates were examined for the formation of a flattened zone of inhibition of the clindamycin disk. Formation of a D shape with erythromycin indicated a positive clindamycin-inducible resistance (iMLSB). Resistance to both clindamycin and erythromycin was recorded as constitutive resistance (cMLSB), and if the isolate was resistant to erythromycin only, it was recorded as macrolide and streptogramin B (MS) phonotype [20].

### 2.3. Ethical Consideration

Ethical approval (Ref. No. 2016-03-11/AEC//Vol.X/190) was obtained from the MUHAS Senate Research and Publications Committee in Dar es Salaam, Tanzania. Permission to conduct the study was sought from participating hospitals. Written informed consent was obtained from each participant.

### 2.4. Data Analysis

Data obtained were analyzed using the Statistical Program for Social Sciences (SPSS) version 20.0. Categorical variables were summarized using proportions. Chi-square tests or Fisher's exact tests were used where applicable to compare differences between proportions. Univariate and multivariate analysis was performed to determine the factors associated with nasal *S. aureus* and MRSA carriage. Odds ratio (OR) and respective 95% confidence interval (CI) were computed. A *p* value < 0.05 was considered as statistically significant.

## 3. Results

### 3.1. Distribution of Sociodemographic Characteristics of Study Participants

A total of 379 HCWs aged between 20 and 61 years were enrolled.  Table 1 shows the distribution of sociodemographic characteristics of the study participants. Most of the subjects were female (222, 58.6%), and majority (325, 85.8%) were aged <45 years. Ocean Road Cancer Institute had a smaller number (19.5%) of participants, while each of the other hospitals contributed almost equal proportions of study participants (26.0%). More than half (203, 53.6%) of the study participants were from medical wards. Of the 379 HCWs, 182 (48%) were medical doctors, while 169 (44.6%) were nurses and the rest (28, 7.4%) were anesthesiologists and radiotherapists. The majority (208, 54.9%) of the HCWs were reported to have been working in their respective hospitals for more than five years. Nearly half (185, 48.7%) of the participants had received antibiotics within the past three months and 42 (11.1%) had history of chronic illness.

### 3.2. Nasal Carriage Rate of *S. aureus* and MRSA

The overall frequency of *S. aureus* nasal carriage among HCWs at the four hospitals was 157/379 (41.4%). Of the 157 *S. aureus* isolates, 59 (37.6%) were MRSA. Therefore, the overall prevalence of MRSA nasal carriage among HCWs was 59/379 (15.6%).  Table 2 shows the characteristics of HCWs according to MRSA versus methicillin-sensitive *S. aureus* (MSSA) carriage status. Of the total number of HCWs colonized with *S. aureus*, 39 (42.1%) female workers were colonized with the MRSA strain. Temeke hospital had a high proportion of HCWs (29, 56.9%), who tested to be MRSA positive, followed by Amana hospital (18, 37.5%). Most (34, 41.9%) of the HCWs who tested to be MRSA positive had history of using antibiotics during the past three months. A high frequency (35, 45.5%) of MRSA was detected among nurses. Other professions including anesthesiologist and radiotherapist had lower MRSA carriage of 3 (25.0%). Thirty-seven (42.5%) HCWs with more than five years of working experience in health care services were colonized with MRSA strains.

### 3.3. Antimicrobial Resistance Pattern

The majority (88, 89.8%) of the *S. aureus* isolates were resistant to penicillin. MRSA isolates showed a significantly high resistance to kanamycin, gentamicin, ciprofloxacin, and trimethoprim-sulphamethoxazole compared to MSSA isolates (*p* ≤ 0.001). All *S. aureus* isolates resistant to both MRSA and MSSA were susceptible to linezolid, while 10.2% of MRSA isolates showed resistance to mupirocin (Table 3). In addition, the proportion of inducible clindamycin resistance (iMLSB) among MRSA isolates was also significantly higher than that among MSSA isolates (*p* ≤ 0.001). Of all the 157 *S. aureus* isolates, 61 (39%) were multiple drug-resistant (MDR) strains, of which 51 (86.4%) were MRSA strains (Table 4).

### 3.4. Factors Associated with MRSA Carriage

Health care workers at Temeke and Amana hospitals had significantly increased odds ratios of acquiring MRSA than those working at ORCI and/or MNH (*p* ≤ 0.001 and 0.005), respectively. Although we did not find significant differences probably due to small numbers, the odds ratio of being colonized with MRSA was higher in nurses compared to the other professions (OR 2.17 (95% CI 0.62–7.63)), in participants with the history of antibiotic use within the past three months (OR 1.4 (95% CI 0.74–2.51)), in HCWs aged >45 years (OR 1.8 (95% CI 0.7–4.4)), and in those working in ICU (OR 1.7 (95% CI 0.7–4.4)). There was no association between MRSA carriage and gender distribution, hand washing, and history of chronic illness among the HCWs investigated (Table 5).

## 4. Discussion

The current study was aimed to determine the prevalence of MRSA nasal carriage among HCWs at tertiary and regional hospitals in Dar es Salaam. The overall prevalence of *S. aureus* colonization among HCWs was 41.4% and that of MRSA carriage was 15.6%, respectively. The nasal carriage of *S. aureus* detected in this study is higher than the rate reported in Kenya (18.3%) [21] and in Northeast Ethiopia (28.8%) [22] but is comparable to that reported in other studies conducted in other developing countries like Botswana (35.8%) [23], Chile (34.9%) [24], and Libya (39%) [25]. We found a high prevalence of MRSA carriage (15.6%) among HCWs from the four hospitals investigated in this study. This prevalence is higher than the previous MRSA carriage reported in the region including 2.1% at MNH in Dar es Salaam [18] and 12.7% in Ethiopia [22]. It is also higher than that reported in the Middle East and Asia, 5.3% in Iran [26], and 3.4% in Nepal [27]. The observed high prevalence in this study compared to others could be due to an overall increase in local MRSA prevalence in patients in the study region over time [28, 29], together with inadequate infection control policies/standards. A study in Nigeria reported a high MRSA prevalence (52.5%) where the author attributed it to lack of infection control policy in the health care facility where the study was conducted [3]. Variation in microbiological methods such as sampling technique sites (nasal, nasopharyngeal, axilla, groin, or web spaces of both hands) and isolation and detection methods might also contribute to the differences observed in various studies [3, 30]. It is also worth noting that the current study included findings from four hospitals in Dar es Salaam, which may have partly influenced the high carriage compared to findings that are based on a study done in one hospital.

In the present study, we found that HCWs working at Temeke and Amana regional hospitals had a significantly higher risk of acquiring MRSA than HCWs at MNH and ORCI. This observation could be explained partly by differences in levels of commitment to control measures of infection between tertiary and regional hospitals suggesting the need for strengthening infection control measures in the regional hospitals in order to prevent spread of MRSA strains. MRSA carriage was particularly high among nurses compared to doctors or other professions. Similar results have been reported in others studies conducted elsewhere [27]. High risk of colonization with MRSA strains among nurses may be due to their frequent patient contact. Preexposure to antibiotics has been reported as a risk factor for MRSA carriage [31]. In the current study, we observed a trend of increased odds ratios of MRSA colonization among HCWs who had a history of using antibiotics in the past three months and in those with >5 years of working experience in the health care services, but the difference was not statistically significant. This is in contrast to findings reported from Northeast Ethiopia where high rates were observed among HCWs with <5 years of working experience [22]. The reason for the observed difference could be due to variation in intensities of exposure to MRSA-colonized HCWs and patients in the two settings.

Frequent hand washing has been reported to reduce the risk of MRSA among HCWs since MRSA strains spread through contaminated hands [32]. Surprisingly, this study failed to demonstrate any significant difference in MRSA carriage between HCWs who frequently wash hands and those who rarely do so after handling patients. This calls for the need to assess hand-washing practices among HCWs in the four hospitals included in this study. Other sociodemographic characteristics such as age and sex or history of chronic illness did not influence the risk of MRSA carriage in this study. This is in contrary to the findings reported from a study conducted in Ethiopia which showed that male HCWs were twice more likely to be MRSA carriers compared to females [22].

Regarding the antimicrobial susceptibility pattern, a high proportion (89.9%) of *S. aureus* isolates were resistant to penicillin. Resistance rates among MRSA isolates toward kanamycin, gentamicin, ciprofloxacin, and trimethoprim-sulphamethoxazole were significantly higher compared to those observed for MSSA isolates. Low resistance rates were detected toward mupirocin and none toward linezolid, indicating that these two drugs can be used as treatment options for MRSA infections in our settings. These findings are similar to those reported in a previous study conducted in the same settings [17]. Low or no resistance could be due to limited prescription of these antibiotics in our hospitals. The resistance observed toward clindamycin was low (13.8%) compared to inducible clindamycin resistance (49.2%) among MRSA isolates, suggesting that testing should be routinely performed in view of the high iMLS_B_ phenotype demonstrated in this study.

Multidrug resistance is currently a worldwide challenge impacting negative consequences in patient management. *S. aureus* is considered MDR if its MRSA strains or the isolates are nonsusceptible to more or equal to one agent in more or equal to three antimicrobial categories [33]. In the present study, MRSA isolates were the predominant MDR strains demonstrated, a finding that is similar to what was reported in a previous study in Tanzania [17].

The limitation in this study was lack of molecular characterization of MRSA strains, which was due to financial constraints, small sample size in some of the variables limiting statistical power, and cross-sectional nature of the design, which affect any conclusion of causation. However, most of the associated factors identified have been studied in a more robust design and found to be biologically and epidemiologically plausible.

## 5. Conclusion

The prevalence of MRSA nasal carriage among HCWs in Dar es Salaam was higher than that previously reported. Nurses in Temeke and Amana regional hospitals had high MRSA carriage. Most of the MRSA isolates were resistant to commonly used antibiotics; however, the rate of resistance to mupirocin and/or linezolid was low, suggesting that these two antibiotics can be used effectively for decolonization or treatment of MRSA infection. Taken together, these findings reinforce the need for more commitment toward implementation of control measures for infection that meet standards in our hospitals including urgent reporting of MRSA laboratory results, assessment of hand hygiene practices, and provision of MRSA education to HCWs. Routine MRSA screening of HCWs should be introduced in health facilities.

## Figures and Tables

**Table 1:** Demographic and clinical characteristics of study participants.

Characteristic	Frequency	Percentage (%)
*Age group (years)*		
<45	325	85.8
>45	54	14.2
*Sex*		
Male	157	41.4
Female	222	58.6
*Hospital*		
Amana	102	26.9
Temeke	104	26.9
MNH	99	26.2
ORCI	74	19.5
*Department/ward*		
Medical wards	203	53.6
Surgical wards	171	45.1
ICU	5	1.3
*Profession*		
Doctors	182	48.0
Nurses	169	44.6
Others^*∗*^	28	7.4
*Duration in health care services*		
<5 years	171	45.1
>5 years	208	54.9
*History of antibiotic use*		
Yes	185	48.7
No	194	51.3
*History of chronic illness*		
Yes	42	11.1
No	337	88.8
*Hand washing*		
Frequently	360	9.1
Occasionally	17	4.5
No	2	0.5

MNH, Muhimbili National Hospital; ORCI, Ocean Road Cancer Institute; ICU, intensive care unit; ^*∗*^anesthesiologist and radiotherapist.

**Table 2:** Characteristics of study participants by MRSA or MSSA carriage status.

Characteristic	MRSA positive (*n*=59), *n* (%)	MSSA (*n*=98), *n* (%)	*p* value
*Age group (years)*			
<45	51 (38.1)	83 (61.9)	0.76
>45	8 (34.8)	15 (65.2)	
*Sex*			
Male	20 (31.2)	44 (68.8)	0.17
Female	39 (41.9)	54 (58.1)	
*Hospital*			
Amana	18 (37.5)	30 (62.5)	0.001
Temeke	29 (56.9)	22 (43.1)	
MNH	10 (27.0)	27 (73.0)	
ORCI	2 (9.5%)	19 (90.5)	
*Department/ward*			
Medical wards	28 (34.6)	53 (65.4)	0.40
Surgical wards	30 (40.5)	44 (59.5)	
ICU	1 (50)	1 (50)	
*Profession*			
Doctors	21 (30.9)	47 (69.1)	0.13
Nurses	35 (45.5)	42 (54.5)	
Others^*∗*^	3 (25.0)	9 (75.0)	
*Duration in health care services*			
<5 years	22 (31.4)	48 (68.6)	0.15
>5 years	37 (42.5)	50 (57.5)	
*History of antibiotic use*			
Yes	34 (42.5)	46 (57.5)	0.19
No	25 (32.5)	52 (67.5)	
*History of chronic illness*			
Yes	4 (22.2)	14 (77.8)	0.15
No	55 (39.6)	84 (60.4)	
*Hand washing*			
Frequently	59 (38.8)	93 (61.2)	0.16
Occasionally	0 (0)	5 (100)	
No	0 (0)	0 (0)	

MNH, Muhimbili National Hospital; ORCI, Ocean Road Cancer Institute; ICU, intensive care unit; ^*∗*^anesthesiologist and radiotherapist.

**Table 3:** Resistance pattern among MRSA and MSSA isolates.

Antimicrobial drug	MRSA (*n*=59), *n* (%)	MSSA (*n*=98), *n* (%)	*p* value
Penicillin	NA	88 (89.8)	
Trimethoprim-sulphamethoxazole	22 (46.8)	5 (9.6)	≤0.001
Ciprofloxacin	42 (71.2)	1 (1.0)	≤0.001
Gentamicin	49 (83.1)	4 (4.1)	≤0.001
Kanamycin	41 (83.7)	6 (6.4)	≤0.001
Clindamycin	8 (13.8)	6 (6.1)	0.1
Erythromycin	46 (78.0)	41 (41.8)	≤0.001
Linezolid^*∗*^	0 (0)	0 (0)	
Mupirocin	6 (10.2)	3 (3.1)	0.06

^*∗*^Antimicrobial susceptibility testing was performed to only 98 *S*. *aureus* isolates. NA, not applicable. MRSA isolates are considered resistant to other beta-lactam agents including penicillin; hence, the testing was not done.

**Table 4:** Prevalence of different antimicrobial resistance types among MRSA and MSSA isolates.

Resistance type	Overall, *n* (%)	MRSA, *n* (%)	MSSA, *n* (%)	*p* value
iMLS_B_	51 (32.5)	29 (49.2)	22 (22.4)	≤0.001
cMLS_B_	18 (11.5)	6 (10.2)	12 (12.2)	0.7
MS phenotype	36 (22.9)	14 (23.7)	22 (22.4)	0.8
MDR	61 (38.9)	51 (86.4)	10 (10.2)	≤0.001
Total	157	59	98	

iMLSB, inducible clindamycin resistance; cMLSB, constitutive clindamycin resistance; MS phenotype, resistance to erythromycin alone; MDR, multidrug resistance.

**Table 5:** Association between MRSA nasal carriage and risk factors among HCWs.

Characteristic	MRSA positive, *n* (%)	Univariate OR (95% CI); *p* value	Multivariate OR (95% CI); *p* value
*Age group (years)*			
<45 (*n*=325)	51 (15.7)	1	1
>45 (*n*=54)	8 (14.8)	1.1 (0.47–2.40); 0.8	1.8 (0.4–4.4); 0.5
*Sex*			
Male (*n*=157)	20 (12.7)	1	1
Female (*n*=222)	39 (17.6)	0.6 (0.38–1.22); 0.2	1.3 (0.57–2.8); 0.5
*Hospital*			
Amana (*n*=102)	18 (17.6)	7.7 (1.73–34.38); 0.007	10.3 (2.0–52.3); 0.005
Temeke (*n*=104)	29 (27.9)	13.9 (3.20–60.47); ≤0.001	20 (3.9–99.3); ≤0.001
MNH (*n*=99)	10 (10.1)	4.04 (0.85–19.05); 0.07	5.3 (1.0–27.9); 0.04
ORCI (*n*=74)	2 (2.7)	1	1
*Department/ward*			
Medical wards (*n*=208)	28 (13.8)	1	1
Surgical wards (*n*=171)	30 (17.5)	0. 64 (0.69–5.93); 0.6	0.7 (0.37–1.32); 0.2
ICU	1 (20)	0.85 (0.92–7.88); 0.8	1.7 (0.15–16.8); 0.7
*Profession*			
Doctors (*n*=182)	21 (11.5)	1.08 (0.30–3.91); 0.8	0. 43 (0.1–1.87); 0.2
Nurses (*n*=169)	35 (20.7)	2.17 (0.62–7.63); 0.2	0. 84 (0.2–3.55); 0.8
Others^*∗*^ (*n*=28)	3 (10.7)	1	1
*Duration in health care services*			
<5 years (*n*=171)	22 (12.9)	1	1
>5 years (*n*=208)	37 (17.8)	0.68 (0.38–1.20); 0.2	2.08 (1.10–4.02); 0.03
*History of antibiotic use*			
Yes (*n*=185)	34 (18.4)	1.52 (0.86–2.66); 0.1	1.4 (0.76–2.58); 0.2
No (*n*=194)	25 (12.9)	1	1
*History of chronic illness*			
Yes (*n*=42)	4 (9.5)	0.54 (0.18–1.57); 0.2	0. 35 (0.11–1.14); 0.08
No (*n*=337)	55 (16.3)	1	1

MNH, Muhimbili National Hospital; ORCI, Ocean Road Cancer Institute; ICU, intensive care unit; ^*∗*^anesthesiologist and radiotherapist.

## Data Availability

All relevant data generated and analyzed during this study are included in this manuscript.
